# The Rho GTPase exchange factor Vav2 promotes extensive age-dependent rewiring of the hair follicle stem cell transcriptome

**DOI:** 10.3389/fcell.2023.1252834

**Published:** 2023-09-26

**Authors:** L. Francisco Lorenzo-Martín, Xosé R. Bustelo

**Affiliations:** ^1^ Molecular Mechanisms of Cancer Program, Centro de Investigación del Cáncer, Consejo Superior de Investigaciones Científicas (CSIC) and University of Salamanca, Salamanca, Spain; ^2^ Instituto de Biología Molecular y Celular del Cáncer, CSIC and University of Salamanca, Salamanca, Spain; ^3^ Centro de Investigación Biomédica en Red de Cáncer (CIBERONC), Salamanca, Spain

**Keywords:** skin homeostasis, aging, epidermal stem cells, hair follicle, gene expression, bioinformatics

## Abstract

Both the number and regenerative activity of hair follicle stem cells (HFSCs) are regulated by Vav2, a GDP/GTP exchange factor involved in the catalytic stimulation of the GTPases Rac1 and RhoA. However, whether Vav2 signaling changes in HFSCs over the mouse lifespan is not yet known. Using a mouse knock-in mouse model, we now show that the expression of a catalytically active version of Vav2 (Vav2^Onc^) promotes an extensive rewiring of the overall transcriptome of HFSCs, the generation of new transcription factor hubs, and the synchronization of many transcriptional programs associated with specific HFSC states and well-defined signaling pathways. Interestingly, this transcriptome rewiring is not fixed in time, as it involves the induction of 15 gene expression waves with diverse distribution patterns during the life of the animals. These expression waves are consistent with the promotion by Vav2^Onc^ of several functional HFSC states that differ from those normally observed in wild-type HFSCs. These results further underscore the role of Vav2 in the regulation of the functional state of HFSCs. They also indicate that, unlike other Vav2-dependent biological processes, the signaling output of this exchange factor is highly contingent on age-dependent intrinsic and/or extrinsic HFSC factors that shape the final biological readouts triggered in this cell type.

## Introduction

HFSCs are essential for the homeostatic balance of the skin (Blanpain and Fuchs, 2006; Blanpain and Fuchs, 2009). They are located in the bulge area of the hair follicle, from which they migrate and progressively differentiate to give rise to the interfollicular, sebaceous, and hair follicle lineages when the skin has to be regenerated due to wounds or other traumas (Blanpain and Fuchs, 2006; Cotsarelis, 2006; Fuchs, 2008; Blanpain and Fuchs, 2009). Consequently, alterations in the number and/or normal functions of these cells contribute to skin regenerative defects, tumorigenic processes, and aging (Schober and Fuchs, 2011; Doles et al., 2012; Hsu et al., 2014). It is therefore of paramount importance to decipher the biological and molecular mechanisms that modulate their numbers, long-term stability, and functional states.

Most Rho GTPases work as molecular switches depending on the type of guanosine nucleotide bound to them (Bustelo et al., 2007). When bound to GDP, these proteins are in an inactive conformation that is not compatible with proximal downstream effector binding. In addition, Rho GTPases are sequestered in the cytosol due to their physical interaction with Rho GDP dissociation inhibitors. However, when in the GTP-bound state, Rho GTPases are unleashed from Rho GDP dissociation inhibitors, interact with a large spectrum of proximal effectors, and eventually promote the stimulation of a wide variety of intracellular functions, such as cytoskeletal remodeling, proliferation, and differentiation (Bustelo et al., 2007). The transition of Rho GTPases from the GDP-bound to the GTP-bound state is catalyzed by GDP/GTP exchange factors (GEF), while the reverse inactivation step is mediated by the GTPase activating proteins (Bustelo et al., 2007). Numerous Rho GTPase have been implicated in regulation of HFSC functions; for instance, Rac1 plays roles in the maintenance of a fully functional HFSC reservoir (Benitah et al., 2005; Chrostek et al., 2006; Castilho et al., 2007; Tscharntke et al., 2007; Chai et al., 2010), RhoA mediates the proliferation and migration of HFSCs (Zhan et al., 2016; Wang et al., 2017), and Cdc42 regulates the fate of epidermal progenitor cells (Wu et al., 2006). More recently, we demonstrated that Vav2, a GEF for Rac1 and RhoA, contributes to regulate the number, responsiveness to stimuli, and functionality of HFSCs (Lorenzo-Martín et al., 2022). This regulation is associated with transcriptional programs that impinge on the proliferation, pluripotency, and quiescence of HFSCs in early postnatal periods (e.g., in 2.5-month-old mice) (Lorenzo-Martín et al., 2022). Consistent with this, we found that the expression of a constitutively active version of Vav2 (referred to hereafter as Vav2^Onc^) in a knock-in mouse model leads to increased numbers of HFSCs and more potent skin regenerative responses upon wound healing or hair depilation (Lorenzo-Martín et al., 2022). The opposite effects are observed in mice lacking Vav2 or the related Vav3 protein. Vav2^Onc^ also changes the transcriptome program of the skin cancer stem cells, including the activation of gene signatures directly associated with the undifferentiated and quiescent state of HFSCs (Lorenzo-Martín et al., 2022).

We have previously shown that the transcriptional program of HFSCs changes quite extensively from early postnatal phases to the adult period in mice (Lorenzo-Martín and Bustelo, 2020). Specifically, we have detected several age-specific gene expression waves associated with functions related to proliferation, differentiation, and aging (Lorenzo-Martín and Bustelo, 2020). These results raise several intertwined questions regarding the impact of Vav2^Onc^ on the HFSC transcriptome: does Vav2^Onc^ promote an age-independent transcriptional program based on its chronically activated state? Alternatively, does Vav2^Onc^ drive a transcriptional program that is subject to age-dependent changes, analogous to those found in normal HFSCs (Lorenzo-Martín and Bustelo, 2020)? If so, does Vav2^Onc^ only promote the amplification of the normal gene expression waves in HFSCs, or does it completely rewire them? To tackle these issues, we have now performed genome-wide expression analyses on HFSCs purified from control or Vav2^Onc^-expressing mice at six different time points of aging (ranging from 18 days to 12 months). By coupling this approach to the use of several *in silico* techniques, we obtained an in-depth overview of the age-dependent dynamics of the transcriptome of HFSCs in both genotypes.

## Materials and methods

### Ethics statement

All mouse experiments were performed according to protocols approved by the Bioethics Committee of the University of Salamanca and the animal experimentation authorities of the autonomous government of Castilla y León (Spain). No patients or patient-derived samples were used in this work.

### Animal studies

Control and *Vav2* ^Onc/Onc^ mice (Fabbiano et al., 2014; Lorenzo-Martín et al., 2020a) had a C57BL/6 J genetic background. Animals were kept in ventilated rooms in a pathogen-free facility under controlled temperature (23°C), humidity (50%) and illumination (12-hour-light/12-hour-dark cycle) conditions.

### Skin stem cell isolation

Hair follicle stem cells were isolated as previously described (Janich et al., 2011). Briefly, mouse back skin was digested in 0.25% trypsin (Thermo Fisher Scientific, cat. # 25200056) overnight at 4°C to collect the keratinocytes from the epidermis. This cell suspension was then filtered, resuspended in EMEM (Lonza, cat. # BE06-174G) supplemented with 15% fetal bovine serum (Thermo Fisher Scientific, cat. # 10500064) and incubated for 30 min on ice with biotin-conjugated antibodies to CD34 (1:50, eBioscience, cat. # 13-0341-85) followed by another 30 min incubation with APC-conjugated streptavidin (1:300, BD Biosciences, cat. # 554067) and PE-conjugated antibodies to CD49f (1:200, AbD Serotec, cat. # MCA699PE). Cells were stained with DAPI (4′,6-diamidino-2-phenylindole) (0.1 ng/μL, Sigma-Aldrich, cat. #D9542) for 5 min to exclude dead cells. Cells positive for CD34 and CD49f were isolated using a FACSAria III flow cytometer (BD Biosciences) and analyzed with the FlowJo software.

### RNA extraction and transcriptome profiling

HFSCs were lysed in RLT buffer (QIAGEN, cat. # 74004), and RNA was extracted using the QIAGEN RNeasy Micro Kit (QIAGEN, cat. # 74004) according to the manufacturer’s instructions. Purified RNA was processed as indicated elsewhere (Menacho-Marquez et al., 2013) and hybridized to Affymetrix GeneChip Mouse Gene 1.0 ST microarrays. R (version 4.1.2). Importantly, WT and *Vav2* ^Onc/Onc^ HFSCs isolation, RNA extraction and transcriptome profiling were performed in parallel. Thus, even though we have already published an in-depth analysis of WT-only HFSC data (Lorenzo-Martín and Bustelo, 2020), the latter is fully comparable with the Vav2^Onc^ dataset reported here. Perl software (version 5.26.2) was used to perform the bioinformatic analyses as previously described (Lorenzo-Martín and Bustelo, 2020). Signal intensity values were obtained from CEL files after applying the Robust Multichip Average (RMA) function from the “affy” package for background adjustment, quantile normalization and summarization (Irizarry et al., 2003).

### Establishment of gene expression patterns

Both Chi–squared and fold-change (FC) thresholds were used to distinguish probe sets with dynamic and stable behavior along the time–points interrogated, as previously reported (Toufighi et al., 2015). Briefly, for each probe set, a chi–squared test with N–1 degrees of freedom were applied as follows:

$$\begin{array}{c} \chi^{2}=\sum_{i}^{n}\frac{{\left(\bar{X_{i}}-\bar{X}\right)}^{2}}{\bar{X}}\\ =\frac{{\left(\bar{X_{0.6}}-\bar{X}\right)}^{2}}{\bar{X}}+\frac{{\left(\bar{X_{1}}-\bar{X}\right)}^{2}}{\bar{X}}+\frac{{\left(\bar{X_{2.5}}-\bar{X}\right)}^{2}}{\bar{X}}+\frac{{\left(\bar{X_{4}}-\bar{X}\right)}^{2}}{\bar{X}}+\frac{{\left(\bar{X_{6}}-\bar{X}\right)}^{2}}{\bar{X}}+\frac{{\left(\bar{X_{12}}-\bar{X}\right)}^{2}}{\bar{X}} \end{array}$$

where $\bar{X_{i}}$ is the mean expression of the gene for the triplicates for each time point *i*, $\bar{X}$ is the overall mean expression across all time points, and *n* is the number of time points. For probe sets satisfying *P* ( $\chi^{2}$ ) < 0.01, a FC ≥ 2 requirement was empirically established. Unsupervised soft clustering was performed using the Mfuzz R package (Kumar and Futschik, 2007); this was used to identify time-course expression profiles. After the expression values were standardized for Euclidian space clustering, the *mfuzz* function with a fuzzifier value of 1.25 and a ranging number of cluster centers was used to determine the optimal number of non-overlapping expression patterns. For probe set inclusion in a particular cluster, the membership value threshold was set to 0.5. The resulting expression profiles were named according to the positions of the main positive (+) and negative (−) enrichments (peaks), i.e., 0.6 = 0.6-month-old; 1 = 1-month-old: 2.5 = 2.5-month-old; 4 = 4-month-old; 6 = 6-month-old; and 12 = 12-month-old. To evaluate how Vav2^Onc^ activity affects HFSC transcriptional dynamics, the fluctuations of each dynamic probe set between the WT genotype (data from (Lorenzo-Martín and Bustelo, 2020)) and the *Vav2* ^*Onc/Onc*^ genotype (data from this study) were analyzed. Using Cytoscape software (Shannon et al., 2003), the results were represented as a network, with nodes indicating expression patterns and edges (arrows) representing how dynamic transcripts behave in the Vav2^Onc^ context as compared to the WT. Node color, color hue, and node size were set according to pattern genotype, connectivity, and size, respectively. The inner ring indicates the dynamic probe sets within the pattern that are not dynamic in the other genotype. Edge thickness positively reflects the amount of transcripts that share the same behavior. The sensitivity for edge representation was set to 10% of the WT pattern size. For visualization of expression and enrichment data in plots, a 0–1 normalization was used as follows:

$$x=\frac{x-x_{\min}}{x_{\max}-x_{\min}}$$

### Gene set annotation and enrichment analyses

Differentially expressed genes between genotypes were identified using linear models for microarray data (limma) (Ritchie et al., 2015), adjusting *p* values for multiple comparisons by applying the Benjamini–Hochberg correction method (Reiner et al., 2003). Functional annotation was performed using Metascape (Tripathi et al., 2015) for biological processes, and ToppFun (Chen et al., 2009) for molecular functions. An FDR q-value of 0.05 was set as threshold for statistical significance. Single-sample gene set enrichment analysis (ssGSEA) (Subramanian et al., 2005; Reich et al., 2006) was used to calculate the fitness of different hallmark, gene ontology, and HFSC signatures across time and genotypes. Hallmark and gene ontology signatures were obtained from the Molecular Signatures Database (Liberzon et al., 2015). The HFSC gene sets were obtained from (Lien et al., 2011). The time-course enrichment scores for these signatures were used to build the signature correlation matrix, calculated through *corrplot* (https://github.com/taiyun/corrplot). Correlations were considered statistically significant when the Pearson coefficient corresponded to *p* ≤ 0.05. Functional clusters were established when every pairwise correlation within a group of signatures was found significant.

### Weighted correlation network analyses

To find the best representatives of every gene expression pattern, the *WGCNA* R package was used (Langfelder and Horvath, 2008). Each weighted gene network was constructed from the corresponding expression matrix using the *blockwiseModules* function. The *pickSoftThreshold* function was used to select the soft thresholding power according to network topology. Consensus module detection within each expression pattern was omitted and kept to one module, as the number of clusters had been already optimized. The heatmap plot depicting the adjacency matrix was created with the *TOMplot* function. To calculate the intramodular connectivity for each gene, the whole network connectivity was computed for each expression pattern through the *intramodularConnectivity* function. Hubs were defined as the 10% most connected genes within each expression pattern. The known functional interactions among hubs were obtained through the String tool (Szklarczyk et al., 2015) and represented using Cytoscape (Shannon et al., 2003). Transcription factor classification was performed using ToppFun (Chen et al., 2009). The iRegulon software was used to determine the transcription factor binding motifs in the promoters of the co-regulated genes (Janky et al., 2014). A collection of 9713 position weight matrices (PWMs) was applied to analyze 10 kb centered around the TSS. With a maximum false discovery rate (FDR) on motif similarity below 0.001, we performed motif detection, track discovery, motif-to-factor mapping, and target detection.

### Statistics

The type of statistical tests performed, and the statistical significance, are indicated for each panel either in the figure legends or in the main text. Data normality and equality of variances were analyzed with Shapiro-Wilk and Bartlett’s tests, respectively. Parametric distributions were analyzed using Student’s t-test. Sidak’s multiple comparison test was used when comparing different sets of means. The chi-squared test was used to determine the significance of the differences between expected and observed frequencies. The Kolmogorov-Smirnov test was used to compare probability distributions. In all cases, values were considered significant when *p* ≤ 0.05. Data obtained are given as the mean ± SEM unless otherwise indicated.

## Data availability

Microarray data corresponding to WT HFSCs were obtained from the GEO database (https://www.ncbi.nlm.nih.gov/geo/) entry GSE137176 (Lorenzo-Martín and Bustelo, 2020). The Vav2^Onc^ data reported in this paper were merged with the former and deposited under the accession number GSE140152.

## Materials availability

All relevant data are available from the corresponding author upon reasonable request. A Materials Transfer Agreement could be required in the case of potential commercial applications.

## Results

### Vav2^Onc^ regulates stem cell transcriptome dynamics

**FIGURE 1:**
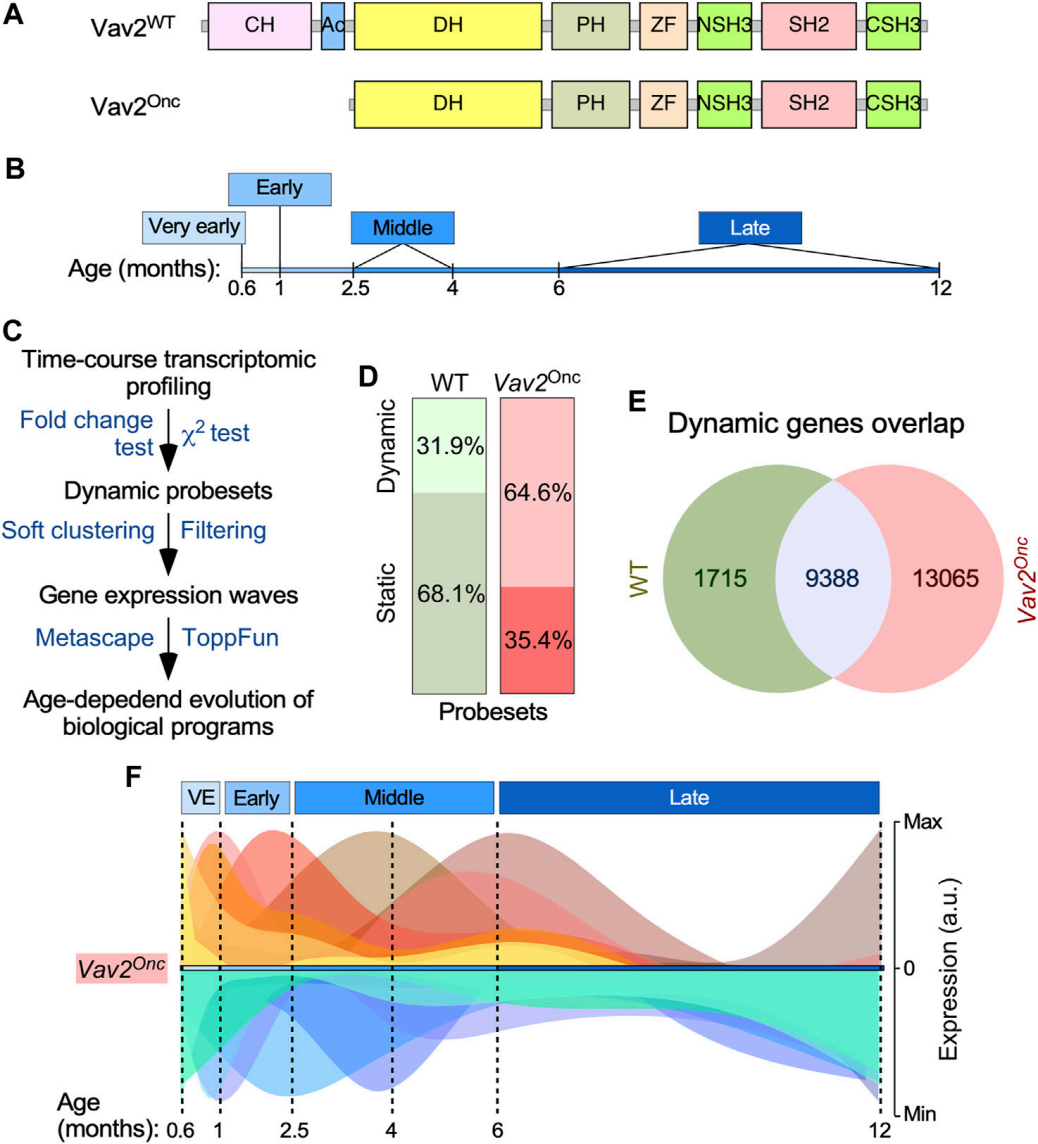
Vav2^Onc^ regulates HFSC transcriptome dynamics **(A)** Scheme of the structure of full-length Vav2 and Vav2^Onc^. Ac, acidic; CH, calponin-homology; CSH3, C-terminal Src-homology 3; DH, Dbl-homology (catalytic domain); NSH3, most N-terminal Src-homology 3; PH, pleckstrin-homology; SH2, Src-homology 2; WT, wild-type; ZF, zinc finger of the C1 subtype. **(B)** Time-windows selected to carry out the microarray analysis using samples from HFSCs. **(C)** Wet-lab and *in silico* pipeline used to identify the time-dependent transcriptome of HFSCs. **(D)** Distribution of the probe sets of the Affymetrix Mouse Gene 1.0 ST array between the dynamic and static groups of HFSCs of indicated genotypes. **(E)** Venn diagram showing the overlap in the dynamic probe sets found in the transcriptome of WT and *Vav2* ^Onc/Onc^ HFSCs. **(F)** Graphical representation of the gene expression waves found in *Vav2* ^Onc/Onc^ HFSCs. The diagram for the gene expression waves of WT HFSCs can be found in (Lorenzo-Martín and Bustelo, 2020).

To investigate the effect of the catalytic hyperactivation of Vav2 in HFSCs, we decided to monitor the time-dependent evolution of the transcriptome of HFSCs isolated from wild-type (WT) and *Vav2* ^Onc/Onc^ knock-in mice. The latter mouse strain, which has been described in previous publications from our lab (Fabbiano et al., 2014; Lorenzo-Martín et al., 2020a), expresses a Vav2 mutant protein (herein, Vav2^Onc^) that exhibits constitutive GEF activity due to the removal of the N-terminal calponin-homology (CH) and acidic (Ac) domains (Figure 1A). These two regions play critical roles in establishing the intramolecular interactions that inhibit the catalytic and most adaptor functions of Vav2 when the protein is not tyrosine-phosphorylated (Schuebel et al., 1998; Zugaza et al., 2002; Bustelo, 2014; Rodriguez-Fdez and Bustelo, 2019). Importantly, the mutant allele that encodes Vav2^Onc^ is under the control of the endogenous *Vav2* promoter, thus ensuring that the mutant protein has a tissue distribution and expression levels comparable to those found in the case of the WT counterpart as demonstrated in previous reports (Fabbiano et al., 2014; Lorenzo-Martín et al., 2020a; Rodriguez-Fdez et al., 2020). As Vav2^onc^ maintains the adaptor functions that are mediated by the C-terminal SH3–SH2–SH3 cassette of the protein (Figure 1A), the effects elicited by its expression must be catalytic-dependent. Consistent with this, we have previously shown that *Vav2* ^Onc/Onc^ mice exhibit phenotypes opposite to those found in mice expressing a catalytically impaired Vav2 protein (L332A mutant) (Lorenzo-Martín et al., 2020b; Rodriguez-Fdez et al., 2020).

We used flow cytometry to isolate HFSCs (CD34^+^ Itgα6^+^) from mice at six time points at different ages (given in months, M), with one time point each classified as very early (0.6-M; 18 days) or early (1-M), and two time points each classified as middle (2.5-M and 4-M) or late (6-M and 12-M) (Figure 1B). The 0.6-M time point corresponds to the earliest stage at which the CD34^+^ Itgα6^+^ population can be clearly identified by flow cytometry (Lorenzo-Martín and Bustelo, 2020). Importantly, WT and *Vav2* ^Onc/Onc^ HFSCs isolation and downstream processing were performed in parallel. Thus, even though we have already published an in-depth analysis of WT-only HFSC data (Lorenzo-Martín and Bustelo, 2020), the latter is fully comparable with the Vav2^Onc^ dataset reported here. In this regard, it is also relevant to note that we did not find any differences in hair cycle staging between WT and *Vav2* ^Onc/Onc^ mice (data not shown).

Genome-wide expression analyses of total RNA isolated from HFSCs were followed by computational approaches specifically designed to identify genes undergoing statistically significant expression changes in at least one of the six time points interrogated (Figure 1C). As previously described (Lorenzo-Martín and Bustelo, 2020), these analyses showed that 31.9% of all the mRNAs expressed in WT HFSCs display a dynamic behavior across time, according to both χ^2^ distribution and expression fold-change (≥2-fold) criteria (Figure 1D, left column) (Lorenzo-Martín and Bustelo, 2020). In contrast, *Vav2* ^Onc/Onc^ HFSCs exhibited a much higher percentage (64.6%) of dynamically regulated genes according to the same selection criteria (Figure 1D, right column). These include 85% of the dynamic transcriptome of WT HFSCs plus an additional subset of 13,065 probe sets that are specific for *Vav2* ^Onc/Onc^ HFSCs (Figure 1E).

**FIGURE 2:**
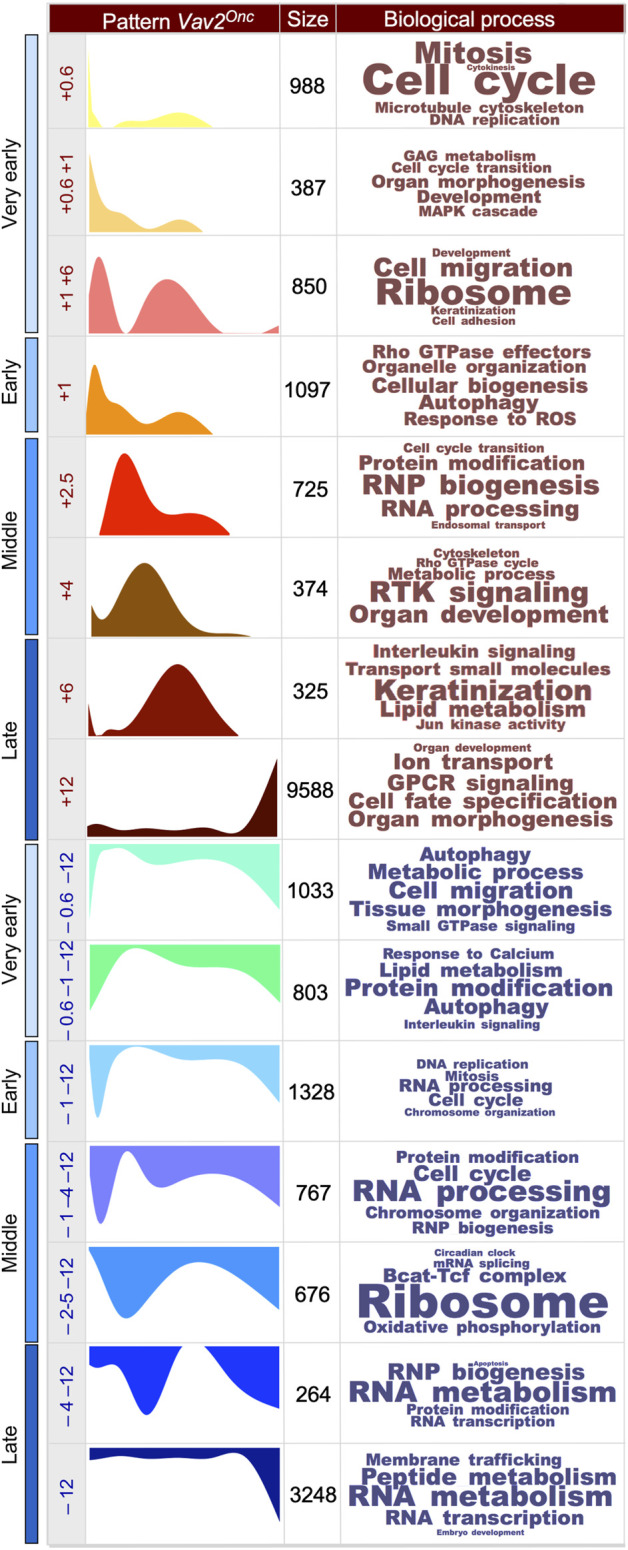
Scheme showing the expression waves found in *Vav2* ^Onc/Onc^ HFSCs. The following information is indicated from left to right: (i) the experimental time point (very early, early, middle, late) in which the indicated gene expression wave is detected; (ii) the time distribution of each wave, given in age of mouse in months (M), of 0.6 M (e.g., 18-days), 1 M, 2.5 M, 4 M, 6 M, and 12 M; (iii) a graphic representation of the indicated gene expression wave along the experimental time points used in this study; (iv) the number of probe sets associated with the indicated gene expression waves; and (v) the top-enriched biological and molecular processes (with font size proportional to the–log(*P*) obtained for each). Each wave is designated as + or − to indicate upregulated (light red waves) or downregulated (light blue waves) genes, respectively. A reference number indicating the time point at which the maximal expression peak of the wave occurs (e.g [+6] or [–12]) is given, whereby numbers for multi-peak waves indicate the highest fold-change peak in the first position (e.g [+1 + 2.5+6] shows that the highest peak was at 1 M). WT HFSCs data can be found in (Lorenzo-Martín and Bustelo, 2020).

We next performed soft clustering to visualize all the dynamically regulated genes that were grouped together in coherent gene expression waves (Figure 1F, see (Lorenzo-Martín and Bustelo, 2020) for WT reference). This was coupled with the use of additional *in silico* tools to allow the identification of the biological processes (GO terms) that were associated with each of those gene expression waves. We found that the HFSCs from WT (14 waves (Lorenzo-Martín and Bustelo, 2020)) and *Vav2* ^Onc/Onc^ mice (15 waves) exhibit similar numbers of time-dependent gene expression waves (Figure 1F, Supplementary Table S1, see (Lorenzo-Martín and Bustelo, 2020) for WT reference). These waves are deconvoluted in Figure 2 to better display the characteristics of each. In this figure, we present the waves classified according to the main time point at which the wave reaches the maximal expression peak value. Each wave is designated with a + or a – symbol depending on whether it contains upregulated (light red waves) or downregulated (light blue waves) genes. In addition, each wave was assigned a reference number that indicates the time point of the maximal expression peak of the wave (e.g [+6] indicates maximal upregulation of gene expression at 6 months). The reference numbers for multi-peak waves include the number of each peak, always placing the highest fold-change peak in the first position) (e.g [+1 + 2.5+6]). Finally, we have included the total number of genes and main GO terms associated with each identified wave (with the size of the letters directly related with the *p*-value of each identified GO term) (Figure 2, see (Lorenzo-Martín and Bustelo, 2020) for WT reference). We found that most of the identified waves in both genotypes have more than one gene expression peak (e.g., waves [+0.6+6] [+2.5+6] [+1+2.3+6] [–1–4], or [–4–12] found in WT HFSCs) (Figure 2, see (Lorenzo-Martín and Bustelo, 2020) for WT reference). The only exceptions to this trend are waves that distribute within the very early time points (e.g., waves [+0.6] and [+0.6+1] in both genotypes) and the late time point (wave [–12] in both genotypes) (Figure 2, see (Lorenzo-Martín and Bustelo, 2020) for WT reference). However, we also found that the waves in WT HFSCs or *Vav2* ^Onc/Onc^ HFSCs are quite different in terms of size (number of genes involved), shape (distribution in time), and encoded functions (Figure 2, see (Lorenzo-Martín and Bustelo, 2020) for WT reference). For instance, we observed that the waves in WT HFSCs contain similar gene content in the time-windows analyzed (very early phase: 3763 probe sets [34.9%]; early phase: 2455 probe sets [22%]; middle phase: 1162 probe sets [10.8%]; late phase: 3405 probe sets [31.6%]) (Figure 2, see (Lorenzo-Martín and Bustelo, 2020) for WT reference). In contrast, in the *Vav2* ^Onc/Onc^ HFSCs, about half of all the dynamically regulated genes are concentrated in the “late” time point (13425 probe sets, 59.6%), while the rest of phases exhibit much lower gene content (very early: 4061 probe sets [18%]; early: 2485 probe sets [11%]; middle: 2542 probe sets [11.3%]) (Figure 2, see (Lorenzo-Martín and Bustelo, 2020) for WT reference). The distribution of the up- and downregulated genes also changes depending on the genotype of the analyzed HFSCs. Accordingly, the ratio of upregulated *versus* downregulated probe sets at the late time point is much higher in *Vav2* ^Onc/Onc^ HFSCs (9913 vs. 3512, 2.8 ratio) than in WT HFSCs (1489 vs. 1916, 0.8 ratio). Inversions in the ratios of up- and downregulated genes are also seen at the very early (1.2 vs. 0.62 ratios) and early (0.82 vs. 1.3) phases between *Vav2* ^Onc/Onc^ HFSCs and WT HFSCs (Figure 2, see (Lorenzo-Martín and Bustelo, 2020) for WT reference). In contrast, the ratios for genes that are mainly regulated in the middle phase are more similar (0.8 and 0.7, for *Vav2* ^Onc/Onc^ HFSCs and WT HFSCs, respectively). Overall, the gene expression waves induced in Vav2^Onc^ HFSCs are more enriched in upregulated genes than those in WT HFSCs. Finally, we have observed that the wave shape differs greatly between WT and *Vav2* ^Onc/Onc^ HFSCs (Figure 2, see (Lorenzo-Martín and Bustelo, 2020) for WT reference). This feature is most conspicuous for the waves of upregulated gene expression at the middle and late time points, which mostly follow a bimodal or a single-peak pattern for WT HFSCs or *Vav2* ^Onc/Onc^ HFSCs, respectively (Figure 2, see (Lorenzo-Martín and Bustelo, 2020) for WT reference). These shape changes can be illustrated by the single peak–enriched waves of *Vav2* ^Onc/Onc^ HFSCs at [+2.5] [+4], and [+6], which are in sharp contrast to the frequently observed bimodal waves of WT HFSCs at [+1+2.5+6] [+2.5+6], and [+6+2.5] (Figure 2, see (Lorenzo-Martín and Bustelo, 2020) for WT reference). Interestingly, this disparity in wave shape is less accentuated at the very early and the late time points (see, for example, the waves [+0.6] [+0.6+1], and [–12] from WT or *Vav2* ^Onc/Onc^ HFSCs; Figure 2, see (Lorenzo-Martín and Bustelo, 2020) for WT reference). The shapes of the waves of downregulated genes are also more similar in the HFSCs of both genotypes (Figure 2, see (Lorenzo-Martín and Bustelo, 2020) for WT reference).

We also observed significant differences in the functions associated with the gene expression waves in the HFSCs of each genotype. Thus, although some functional overlap is seen at specific time points (e.g., the cell growth, ribosomal biosynthesis, autophagy-related functions, and inflammatory-related functions in waves [+0.6] [+0.6+6] [+12], and [+6+2.5]) (Figure 2, see (Lorenzo-Martín and Bustelo, 2020) for WT reference), the spectrum of wave-associated functions is highly genotype-dependent (Figure 2, see (Lorenzo-Martín and Bustelo, 2020) for WT reference). In a few cases, the same functions are detected at different time windows (e.g., ion transport is in wave [+2.5+6] in WT HFSCs but in wave [+12] in *Vav2* ^Onc/Onc^ HFSCs; keratinocyte differentiation is in wave [+1+2.5+6] in WT HFSCs but in wave [+6] in *Vav2* ^Onc/Onc^ HFSCs). Collectively, these results indicate that: 1) Vav2^Onc^ promotes a significant rewiring of the age-dependent transcriptional program of HFSCs in terms of total gene numbers, time distribution of the waves of differentially expressed genes, and overall functions targeted; and 2) even though Vav2^Onc^ is chronically activated in a cell stimulus-independent manner, it does not engage a fixed transcriptional program in HFSCs. This suggests that, in addition to the intrinsic catalytic activity, the impact of Vav2^Onc^ on HFSC biology is also influenced by other time-dependent intrinsic or extrinsic factors that contribute to shaping its impact on the transcriptome of HFSCs.

Analyzing the functions associated with the gene expression waves also shed further light on the impact of Vav2^Onc^ on HFSC biology. Thus, during the very early time point, the upregulated gene expression waves of WT HFSCs encode functions related to cell growth, IGF (insulin growth factor) and mTORC signaling, extracellular matrix interaction regulators, and several metabolic routes. This spectrum of functions is probably associated with the expansion in the numbers of HFSCs that is usually observed between postnatal days 18 and 30 in mice (Lorenzo-Martín and Bustelo, 2020). In contrast, the waves associated with downregulation events contain genes encoding proteins involved in small GTPase signaling, cell adhesion, protein modification, and autophagy. This program is substituted as the animals age by the upregulation of genes involved in catabolic (e.g., autophagy, ubiquitination), cell differentiation, cell adhesion, and inflammatory processes (Figure 2, see (Lorenzo-Martín and Bustelo, 2020) for WT reference). In parallel, we observed downmodulation of genes linked to cell cycle regulation, receptor tyrosine kinase (RTK) signaling, ribogenesis, and metabolic programs (TCA cycle, sterol, and small molecule biosynthesis) (Figure 2, see (Lorenzo-Martín and Bustelo, 2020) for WT reference). This pattern can be linked to the post-mitotic telogen phase of the skin (which takes place in approx. 2.5-month-old mice) and with the subsequent acquisition of aging features by HFSCs at later stages of the time-course analyzed in this study (Doles et al., 2012; Lorenzo-Martín and Bustelo, 2020). A comprehensive description of the time-dependent evolution of all these functions can be found elsewhere (Lorenzo-Martín and Bustelo, 2020). In the case of Vav2^Onc^ HFSCs, we find similar, significantly amplified functional features related to cell growth upregulation at the very early time point (Figure 2, see (Lorenzo-Martín and Bustelo, 2020) for WT reference). In contrast, the downregulation of cell growth-related genes that takes place at the early time point is less acute in these cells than in the controls (Figure 2, see (Lorenzo-Martín and Bustelo, 2020) for WT reference). At later time points, the upregulated waves of Vav2^Onc^-expressing HFSCs include a large variety of functions not seen in the WT counterparts, such as signaling pathways for MAPK [+0.6+1], RTK [+2.5+6], Rho GTPase [+2.5+6], Jun-N-terminal (JNK) [+6+2.5], G-coupled protein receptors (GPCR) [+12], and ribonucleoprotein (RNP) biogenesis [+1+2.5+6] (Figure 2, see (Lorenzo-Martín and Bustelo, 2020) for WT reference). This indicates that, from a signaling, metabolic, and developmental perspective, the *Vav2* ^Onc/Onc^ HFSCs fluctuate between functional states during the time-courses analyzed, and that these fluctuations are quite different from those found in WT HFSCs except at the very early time point. The functions associated with the waves of gene downregulation are also quite different between WT and *Vav2* ^Onc/Onc^ HFSCs (Figure 2, see (Lorenzo-Martín and Bustelo, 2020) for WT reference). The most conspicuous functional change is the downmodulation of RNA- and ribosomal-related functions in *Vav2* ^Onc/Onc^ HFSCs starting at the middle time points (Figure 2, see (Lorenzo-Martín and Bustelo, 2020) for WT reference). Thus, these data indicate that Vav2^Onc^ induces a large rewiring of the gene expression waves at the level of total gene numbers, time-dependent distribution of the waves, and functions involved. They also suggest that *Vav2* ^Onc^ favors the stepwise engagement of different HFSC functional states that are not usually seen in WT HFSCs at most time points.

### Vav2^Onc^ reshapes the time-dependent expression waves present in skin stem cells

**FIGURE 3:**
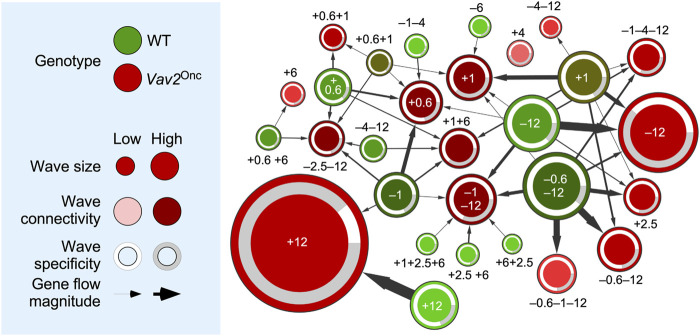
Vav2^Onc^ reshapes the gene expression waves found in HFSCs. *Left*, summary of the parameters used in the network shown on the right. *Right*, dynamic flow network showing the relationship between WT (green) and *Vav2* ^Onc/Onc^ (red) gene expression waves (nodes). Each expression wave is identified by the position (0.6-to-12 months old) and orientation (upregulated [+] or downregulated [–]) of its main peak(s) indicated in Figure 2. Wave size (number of probe sets), connectivity (number of interacting clusters in terms of probe set sharing), specificity (number of probe sets that are not dynamic in the other genotype), and flow magnitude (number of probe sets that are shared between two clusters) are indicated.

Based on the extensive transcriptomal rewiring observed in the foregoing experiments, we decided to build a network map to better visualize the changes in wave localization of the age-regulated genes in the case of WT and *Vav2* ^Onc/Onc^ HFSCs. In this map, the gene expression waves found in WT and *Vav2* ^Onc/Onc^ HFSCs are depicted as green and red circles, respectively (Figure 3). In both cases, the size and darkness of the color of each wave are proportional to the total number of dynamically regulated probe sets of each wave and to the level of changes in gene probe localization seen between the WT and *Vav2* ^Onc/Onc^ conditions, respectively (Figure 3). The change in position of gene probes is further highlighted by the thickness of the arrows connecting the indicated WT and *Vav2* ^Onc/Onc^ HFSC waves (Figure 3). Finally, the circles also include information on the percentage of genes that are shared with waves of the opposite genotype (white peripheral areas) or of those that are just seen in the waves of a single genotype (gray peripheral areas) (Figure 3). Importantly, we also include the reference numbers assigned to the waves in Figure 2 to facilitate their identification in Figure 3.

These analyses revealed extensive repositioning of transcripts between the WT and *Vav2* ^Onc/Onc^ HFSC waves. This phenomenon is quite significant for the *Vav2* ^Onc/Onc^ waves at: 1) [+0.6], which also contains transcripts present in the WT waves [–1], [+0.6], [+0.6+1], [–1–4] and [+1]; 2) [+1], with transcripts present in the WT waves [+1], [+0.6+1], [–6], and [–12]; 3) [+1+6], with transcripts present in the WT waves [+0.6], [–1], [–1–4] [–12], and [–1–12]; and 4) [–1–12], with transcripts present in the WT waves [+1+2.5+6], [+2.5 +6], [+6+2.5], [–0.6–12], [–1], and [–12] (Figure 3). The *Vav2* ^Onc/Onc^ waves that were less affected with this reshuffling in the position of dynamically regulated genes include those at: 1) [+6], [–0.6–1–12], and [–4–12], whereby changes in position affected just one wave of each genotype; and 2) [+0.6+1] and [–0.6–12], whereby changes in position involved two waves (Figure 3). The *Vav2* ^Onc/Onc^ wave [+4] is the only one that does not exhibit any gene originally with respect to the WT waves (Figure 3). This wave is mainly composed of genes associated with RTK signaling, Rho GTPase signaling and functions, organ development, and metabolism (Figure 2, see (Lorenzo-Martín and Bustelo, 2020) for WT reference). Conversely, the WT waves [–0.6–12] and [+1] contained more genes that changed position in the *Vav2* ^Onc/Onc^ waves (with seven and six inter-wave changes in localization, respectively) (Figure 3). On the other hand, the WT waves with lower levels of gene changes include [+1+2.5+6] [+2.5+6] [+6+2.5] [–6] [–1–4], and [–12] (with a single inter-wave change in localization for each), although some content changes involved substantial numbers of transcripts (e.g., WT wave [+12]) (Figure 3). These changes in wave localization are not unidirectional, as subsets of mRNAs belonging to a specific WT wave can be found in different *Vav2* ^Onc/Onc^ HFSC waves (Figure 3). For example, the transcripts present in the WT wave [–1] are found in five different *Vav2* ^Onc/Onc^ waves ([+0.6], [+1+6], [+12], [–2.5–12], and [–1–12]) (Figure 3).

Importantly, the genes involved in this transcriptional rewiring might not necessarily keep the same expression pattern in the two genotypes (e.g., genes in the upregulated WT waves [+1+2.6+6] [+2.5+ 6] and [+6+2.5] are found in the downregulated *Vav2* ^Onc/Onc^ HFSC wave [–1–12], while genes in the downregulated WT HFSC wave [–6] are found in the upregulated *Vav2* ^Onc/Onc^ wave [+1]) (Figure 3). In other cases, different gene subsets of the same WT wave display the same and the opposite regulation in the *Vav2* ^Onc/Onc^ waves (e.g., genes in the WT wave [+1] that become located in *Vav2* ^Onc/Onc^ waves [+1], [+2.5], [+1+6], [–0.6–12], [–4–12], [–1–4–12] and [–12]) (Figure 3). We have also found that this rewiring is highly dependent on the time-window involved. Thus, most of the *Vav2* ^Onc/Onc^ waves that are distributed between the very early and middle time points contain genes that are also dynamically regulated under normal physiological conditions in WT HFSCs (Figure 3, see white areas in the peripheral band of each wave). In contrast, at the late time point, the *Vav2* ^Onc/Onc^ waves become enriched in genes that are not found dynamically regulated in the WT waves (Figure 3, see grey areas in the peripheral band of each wave). In fact, we estimated that ≈60% of all the dynamically regulated genes found in *Vav2* ^Onc/Onc^ HFSCs are non-dynamic in the case of WT HFSCs at this time point (e.g., see *Vav2* ^Onc/Onc^ HFSC waves [+12] and [–12]) (Figure 3). These data suggest that Vav2^Onc^ first promotes an extensive rewiring of the transcriptional program of HFSCs and, subsequently, the emergence of transcriptional programs that are mostly Vav2^Onc^-dependent. This is associated with a total change in type and/or temporal distribution of biological processes in Vav2^Onc^-expressing HFSCs.

### Vav2^Onc^ promotes the synchronization of pathway-associated gene signatures

**FIGURE 4:**
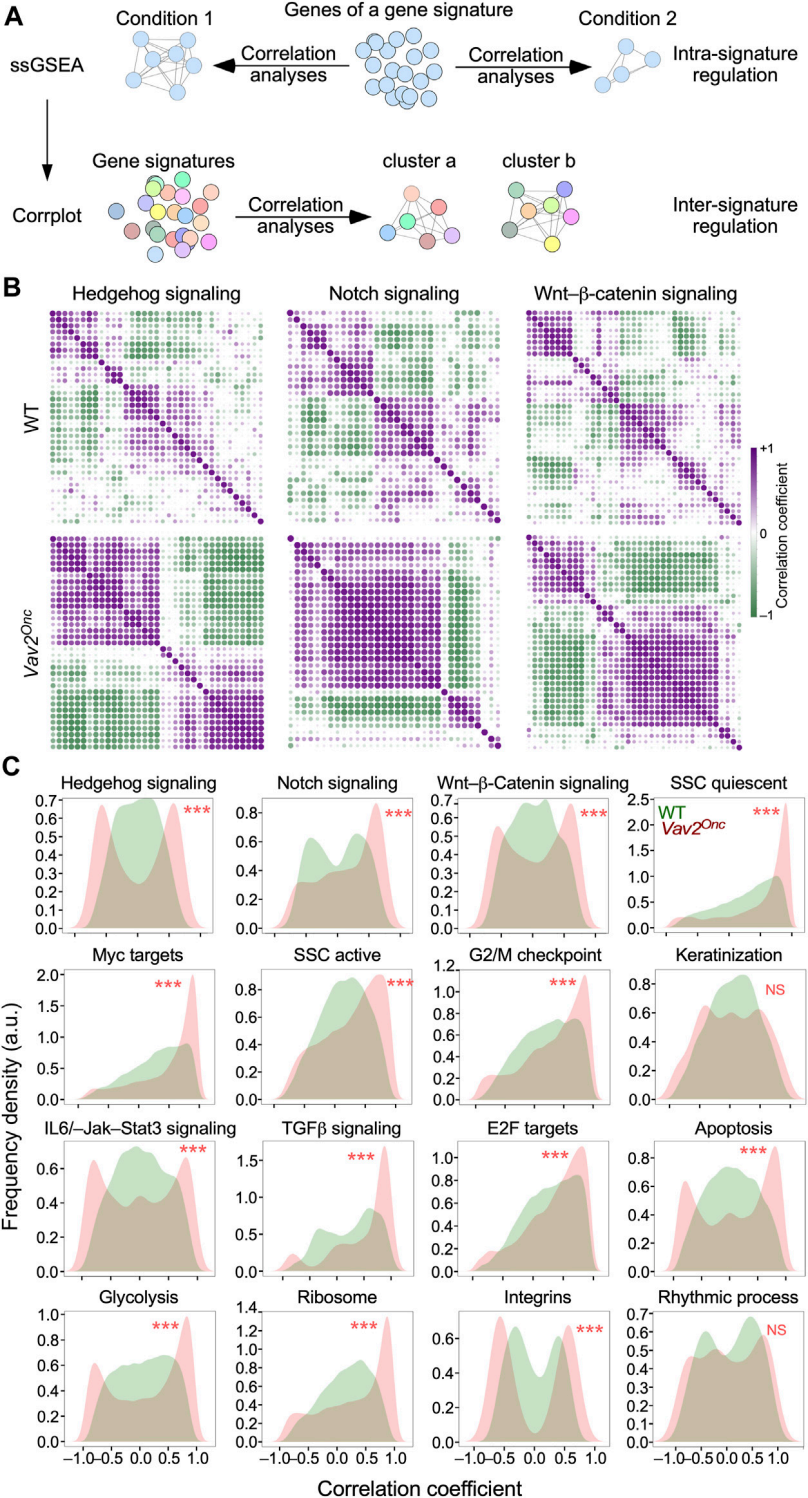
Vav2^Onc^ promotes the synchronization of pathway-associated gene signatures **(A)** Scheme of the *in silico* analyses performed to identify intra-signature (top) and inter-signature (bottom) coregulatory events in the transcriptome of HFSCs. See main text for further details. **(B)** Co-expression matrices of the transcripts integrating the indicated gene signatures in WT (top) and *Vav2* ^Onc/Onc^ (bottom) HFSCs. The *r* value of a given pair of gene signature elements is shown as a purple or a green dot if the two genes are co-regulated (*r* > 0) or anti-regulated (*r* < 0), respectively. Dot size and color gradient reflects the magnitude of the Pearson correlation coefficient (*r*). **(C)** Density plots showing the distribution of *r* values in the coexpression matrices of indicated gene signatures in WT (green) and *Vav2* ^Onc/Onc^ (red) HFSCs. ***, *p* < 0.001 (Kolmogorov-Smirnov test). NS, not significant.

We have previously shown that the time-dependent evolution of the transcriptome of WT HFSCs is associated with specific patterns of co-regulation of genes signatures that are linked to well-defined hallmarks and HFSC-related biological and signaling processes (Lorenzo-Martín and Bustelo, 2020). This led us to: **i)** investigate the impact of chronic Vav2^Onc^ signaling on the regulatory pattern of such gene signatures; and **ii)** focus on more specific functions rather than on those usually associated with the very generic GO terms used to classify the expression waves in Figure 2. To this end, we utilized the transcriptomal data obtained from WT and *Vav2* ^Onc/Onc^ HFSCs to carry out two concatenated *in silico* steps. First, we performed co-expression analyses to identify patterns of co-regulation of the gene elements of each interrogated gene signature throughout all time points used in our study (Lien et al., 2011; Liberzon et al., 2015) (Figure 4A, top). In these analyses, we can expect different types of distributions based on the obtained correlation coefficients *r*): Type 1, distributions with *r* centered around r ∼ 0, which indicates that the elements a gene signature do not follow stable coexpression trends; type 2 and 3, distributions skewed towards *r* > 0 (type 2) or *r* < 0 (type 3), indicating that a significant fraction of the genes is transcriptionally coregulated in one direction (positive or negative correlation); and, Type 4, bimodal distributions with *r* values both above and below 0, which indicate that subsets of genes of the same signature follow opposite expression patterns. The co-regulation of all gene elements of a given signature can be plotted using co-expression matrices, such as those shown in Figure 4B. Finally, we used single-sample gene set enrichment analyses (ssGSEA) for all the interrogated signatures and built co-expression matrices to pick up clusters of co-regulated or anti-regulated gene signatures in the transcriptome of WT and *Vav2* ^Onc/Onc^ HFSCs (Figure 4A, bottom).

In the first step, we found that the elements (single genes) of most interrogated gene signatures follow a type 1 distribution (*r* ≈ 0) (Figure 4B, upper panel; Figure 4C, green graphs) and, to a lesser extent, type 4 bimodal patterns (Figure 4C, green graphs) in WT HFSCs. Examples of each of those distributions include the hedgehog and integrin gene signatures, respectively (Figure 4C, green graphs). In contrast, for *Vav2* ^Onc/Onc^ HFSCs, we observed a massive increase in the overall degree of co-regulation and/or anti-regulation of the elements of most gene signatures (Figure 4B, lower panels). This is due to the enhancement of either the co-regulation or anti-regulation previously seen in the time-dependent transcriptome of WT HFSCs (Figure 4B, compare the upper and lower levels). Due to this, the co-expression patterns found in *Vav2* ^Onc/Onc^ HFSCs show in most cases a stronger polarization towards the type 2 (r > 0) and type 4 (r ≈ ±1) distributions (Figure 4C, red graphs). Examples of each of those distributions are the elements of the epidermal stem cell quiescent signature and the hedgehog signaling signature, respectively (Figure 4C, red graphs). This is a general phenomenon, as it is observed in 14 of the 16 gene signatures interrogated in these analyses (Figure 4C, red graphs). The only exceptions to this trend are the gene signatures linked to keratinization and rhythmic processes (Figure 4C, red graphs). These data indicate that the expression of Vav2^Onc^ increases the synchronization of gene expression of most functional gene signatures interrogated in this study.

**FIGURE 5:**
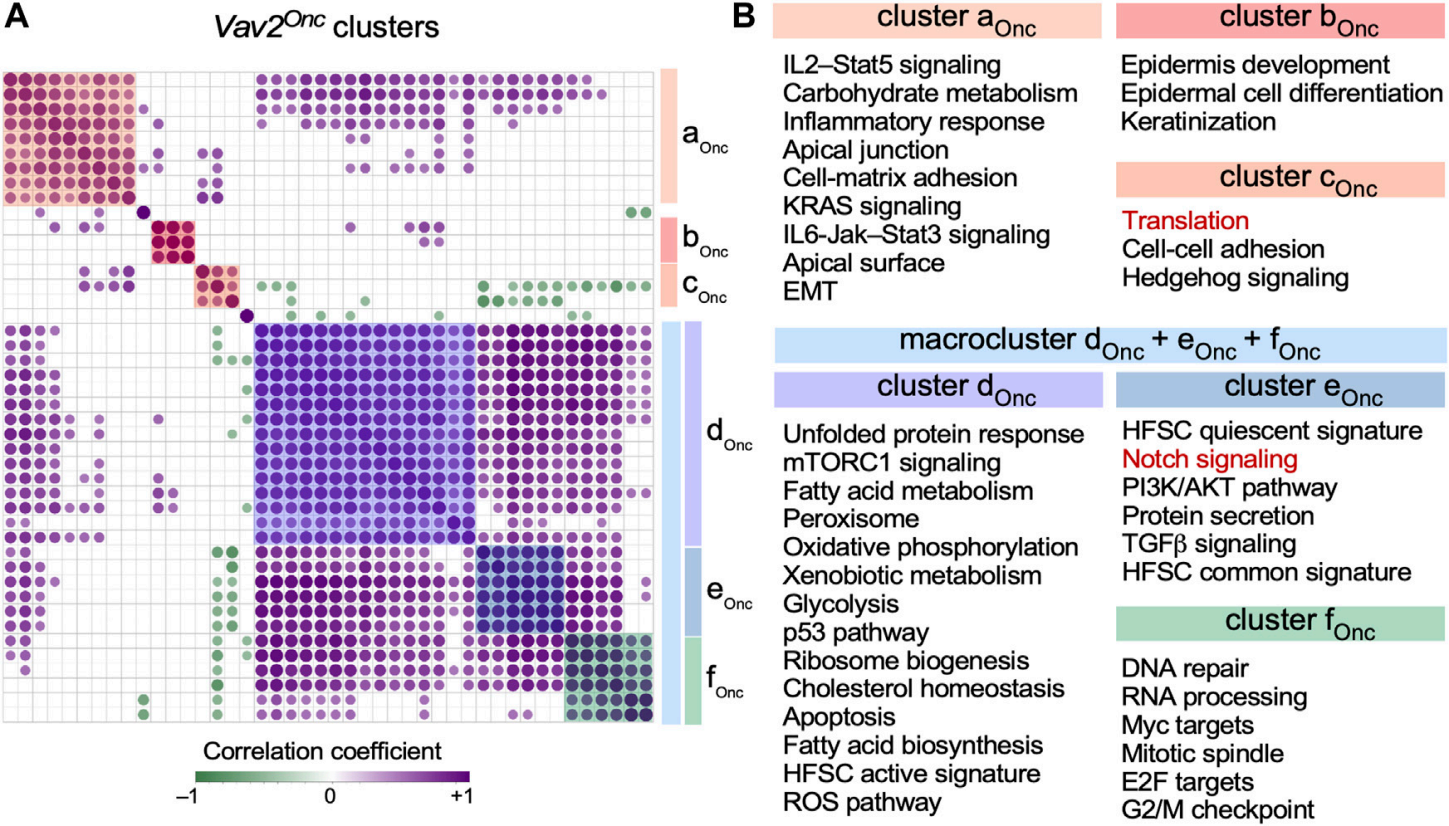
Vav2^Onc^ promotes synchronization of multiple gene signatures in HFSCs **(A)** Co-expression matrix showing the correlation across time among the enrichment scores of the gene expression signatures shown in **(B)** in *Vav2* ^Onc/Onc^ HFSCs. Only correlations with *p* ≤ 0.05 have been considered as statistically significant. Positive and negative correlations are shown in purple and green, respectively. Dot size and color gradient reflects the magnitude of the r value obtained for each indicated pair. Pathway clusters are indicated with color shades. The co-expression matrix for the WT HFSC gene signatures can be found in (Lorenzo-Martín and Bustelo, 2020). **(B)** Gene signatures co-regulated in the same clusters in *Vav2* ^Onc/Onc^ HFSCs. Red indicates gene signatures that are not conserved in the clusters of the opposite genotype HFSCs.

Strikingly, using ssGSEA (Figure 4A, bottom panel), we observed that Vav2^Onc^ also favors a very strong synchronization of the expression of several independent gene signatures (Figures 5A, B). Consistent with this, the 10 inter-gene signature clusters previously described in WT HFSCs (Lorenzo-Martín and Bustelo, 2020) become consolidated into just 3 clusters and a large macrocluster in *Vav*2^Onc/Onc^ HFSCs (Figures 5A, B). As in our previous analyses (Figure 3), we observed an extensive reshuffling of the interrogated gene signatures from specific clusters of WT HFSCs to the new clusters found in *Vav2* ^Onc/Onc^ HFSCs (Figure 5A, see (Lorenzo-Martín and Bustelo, 2020) for WT reference). In fact, there is only one cluster of co-regulated gene signatures that is identical in the HFSCs of both genotypes (namely, clusters c_WT_ and b_Onc_; Figure 5, see (Lorenzo-Martín and Bustelo, 2020) for WT reference). This cluster harbors co-regulated gene signatures associated with epidermal development, epidermal cell differentiation, and keratinization (Figure 5B). In addition to this extensive reshuffling, we have also found very limited cases of either loss or gain of new co-regulated gene signatures in the clusters found in *Vav*2^Onc/Onc^ HFSCs. Those include the loss of the Wnt–β-catenin and TNFα–NFκB signaling gene signatures (Figure 5B, see (Lorenzo-Martín and Bustelo, 2020) for WT reference) and the *de novo* incorporation of a protein translation-associated gene signature to the cluster designated as c_Onc_ in *Vav*2^Onc/Onc^ HFSCs (Figure 5B, red font).

### Vav2^Onc^ rewires the transcription factor landscape in skin stem cells

**FIGURE 6:**
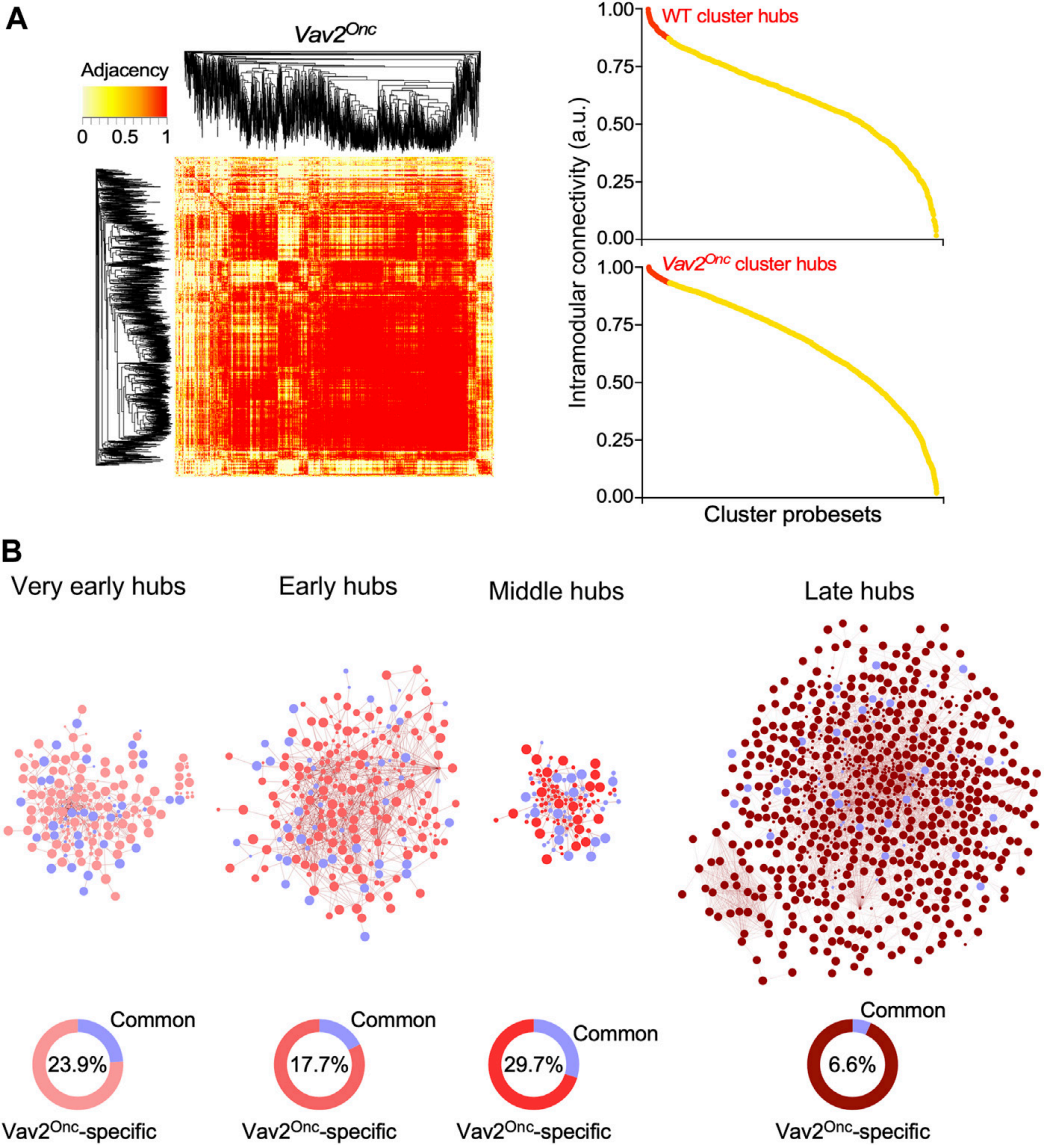
Vav2^Onc^ promotes new transcriptomal hubs in HFSCs **(A)** *Left*, representative example of the adjacency heatmaps computed for each expression pattern applying the WGCNA algorithm to the *Vav2* ^Onc/Onc^ HFSC transcriptome (see (Lorenzo-Martín and Bustelo, 2020) for WT reference). Red areas indicate the presence of probe sets that are highly connected transcriptionally. Probe set clustering according to adjacency is depicted on the sides of the heatmap. *Right*, representative examples of dot plots showing the intramodular connectivity score for each of the probe sets belonging the coexpression clusters analyzed in the right panel. Transcriptional hubs (highlighted in red) are defined as the 10% most interconnected probe sets. **(B)** *Top*, transcriptomal hubs found in *Vav2* ^Onc/Onc^ HFSCs at the indicated experimental time points. The hubs shared with WT are shown in blue color. *Bottom*, percentage of hubs that are specific to *Vav2* ^Onc/Onc^ HFSCs (red) or common to both *Vav2* ^Onc/Onc^ and WT HFSCs (blue) (blue). The percentage of hubs shared by HFSCs of both genotypes is given inside each graph.

We assumed that the extensive age-dependent transcriptional rewiring observed in *Vav2* ^Onc/Onc^ HFSCs had to be associated with changes in the normal transcription factor landscape of skin stem cells. To investigate this, we next performed weighted correlation network analyses (WGCNA) to calculate hierarchical co-expression relationships among all the genes identified in the expression waves found in both WT and mutant HFSCs. This approach entailed the calculation of the adjacency and the intramodular connectivity for all the wave gene components (Figure 6A, see (Lorenzo-Martín and Bustelo, 2020) for WT reference), two parameters that are highly related to the Eigengene-based cluster membership score of each transcript. To further increase the stringency of these analyses, we defined hubs as only the 10% of the top-connected nodes in each age period-specific gene expression wave (Figure 6A, right panels). We found that only 26.5% of the transcriptomal hubs are conserved between WT and *Vav2* ^Onc/Onc^ HFSCs, which is consistent with the extensive transcriptomal rewiring observed in Figures 2–4. This reduced overlap is seen at the very early (23.9% of shared hub elements), early (17.7% of shared hub elements), middle (29.7% of shared hub elements), and late (6.65% of shared hub elements) experimental time points (Figure 6B, blue colors). The very low level of overlap in the hub elements between WT and *Vav2* ^Onc/Onc^ HFSCs seen in the late time-window is consistent with the increase of Vav2^Onc^-specific transcriptional programs previously observed in our *in silico* analyses (Figure 2; Figure 3). These findings indicate that the overall transcriptional rewiring induced by Vav2^Onc^ in HFSCs is accompanied by a parallel reshaping of the overall transcriptomal hub landscape.

**FIGURE 7:**
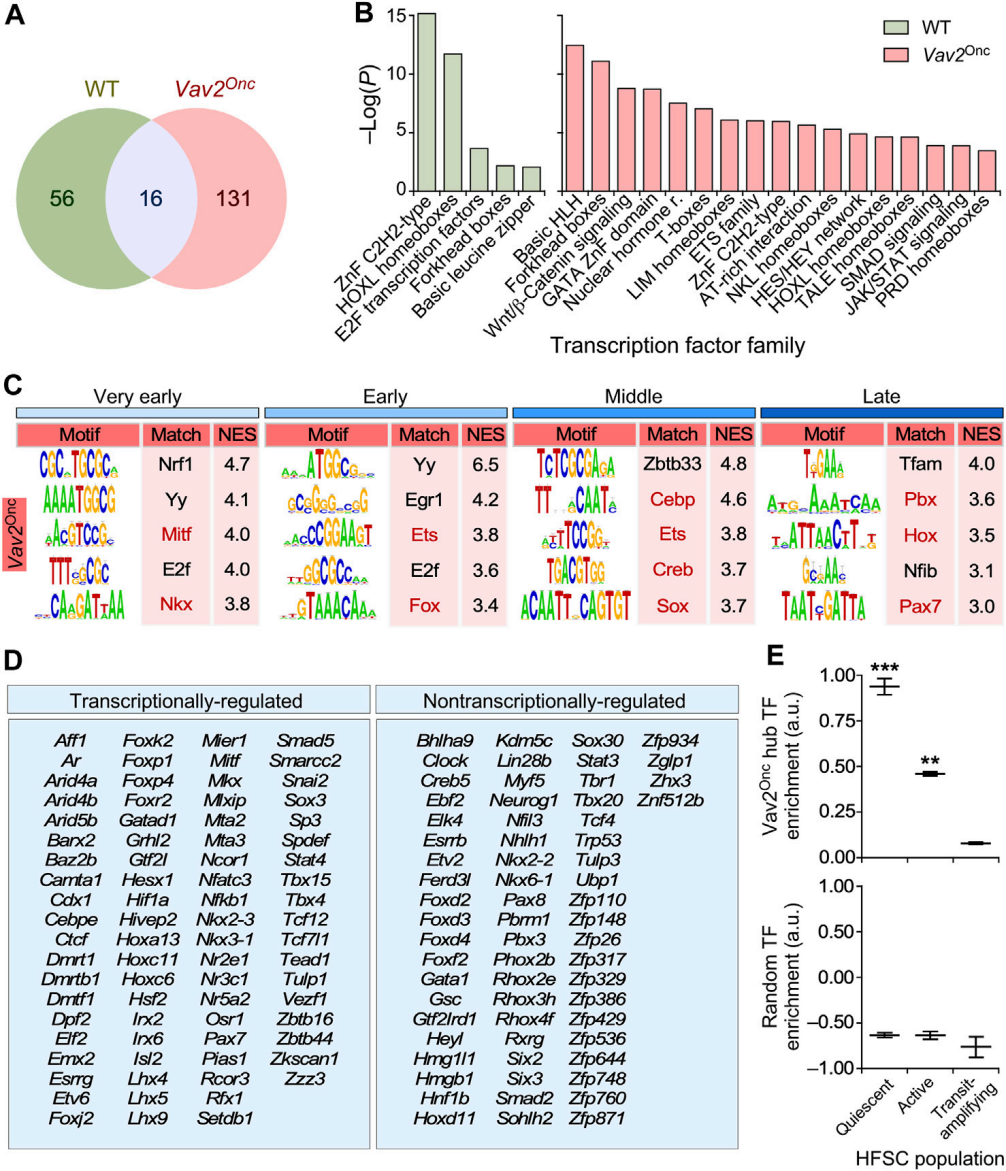
Vav2^Onc^ rewires the transcription factor (TF) landscape in HFSCs **(A)** Venn diagram showing the overlap in TF identify in the transcriptomal hubs of WT or *Vav2* ^Onc/Onc^ HFSCs. **(B)** Main TF families enriched in the WT or Vav2^Onc^ collections of TF hubs. For all, *p* ≤ 0.05 (Fisher’s exact test). **(C)** Main enriched TF binding sites in the promoters of time-regulated genes from *Vav2* ^Onc/Onc^ HFSCs (see (Lorenzo-Martín and Bustelo, 2020) for WT reference). Red indicates sites for which the TF is in a Vav2-regulated transcriptional hub. Normalized enrichment scores (NES) are indicated. For all, *p* ≤ 0.05 (iRegulon Wilcoxon rank-sum paired test). **(D)** List of TFs found in the transcriptomal hubs of *Vav2* ^Onc/Onc^ HFSCs. TFs that are differentially expressed at any time point as compared to WT HFSCs are labeled as “transcriptionally-regulated” (left column). **(E)** Plots showing the enrichment of the Vav2^Onc^ hub TFs (top) and of an equally sized random collection of TFs (bottom) in the indicated epidermal cell populations. **, *p* < 0.01; ***, *p* < 0.001 against TAC population (Student’s t-test). Data represent median, minimum, and maximum values. TAC, transit-amplifying cell.

We hypothesized that the transcription factors responsible for the Vav2^Onc^-mediated transcriptomal rewiring had to be contained within the hubs described above. In line with this idea, we found that the landscape of transcription factors acting as hubs was very different between WT and *Vav2* ^Onc/Onc^ HFSCs (Figure 7A). In WT HFSCs, they include members of the zinc finger C2H2, HOX-like, and E2F families (Figure 7B, Supplementary Table S2). In contrast, in *Vav2* ^Onc/Onc^ HFSCs, they encompass specific members of the basic helix-loop-helix, Forkhead box, Gata, nuclear hormone receptor, Tcf, Ets, Nkl, Hox-like, Stat, and pluripotency-associated families (Figure 7B, Supplementary Table S2). We reasoned that if these transcription factors mediate the Vav2^Onc^-induced transcriptomic remodeling, they had to target many of genes present in the Vav2^Onc^-driven expression waves. Consistent with this idea, we found that most of the dynamically regulated genes in *Vav2* ^Onc/Onc^ HFSCs (Figure 2) harbor site(s) for these transcription factors (Figures 7B, C; Supplementary Table S2). This is Vav2^Onc^-specific, since these sites are not enriched in the case of the dynamically regulated genes in WT HFSCs (Supplementary Table S2). Indeed, our microarray data indicate that Vav2^Onc^ transcriptionally regulates the activity of a large fraction (55%) of these transcription factors (Figure 7D, left panel). Importantly, ssGSEA analyses indicate that these Vav2^Onc^-driven transcription factors show high expression levels in quiescent HFSCs and become strongly downmodulated upon SCC activation and differentiation (Figure 7E, top panel). As a control, performing these analyses using equally sized random collections of transcription factors shows no significant enrichments (Figure 7E, bottom panel).

## Discussion

Given their stemness condition, it could be inferred that HFSCs have to remain in a “platonic-like”, stable functional state throughout the life of an organism unless they are required for regenerative responses. However, we have recently shown that approximately one-third of the transcriptome of mouse HFSCs undergoes quite significant age-dependent variations under normal tissue homeostatic conditions. This is associated with well-defined age-dependent gene expression and functional waves (Lorenzo-Martín and Bustelo, 2020). Likewise, an independent study has demonstrated extensive changes in the transcriptome of adult (18-month-old) HFSCs (Doles et al., 2012). It is likely that these transcriptome changes are dependent on internal HFSC clocks as well as on external signals from the surrounding cells of the HFSC bulge niche. In addition to the time dimension, the functional features of HFSC are also highly influenced by the activation state of specific signal transduction proteins. Thus, we have shown in a recent study that interfering with the catalytic function of Vav2, using either a gain-of-function or a loss-of-function approach leads to extensive changes in the numbers, regenerative capacity, and the overall transcriptome of HFSCs isolated from 2.5-month-old mice. Such changes are connected to increasing proliferation while maintaining the biological programs linked to HFSC pluripotency (Lorenzo-Martín et al., 2022). However, given that these analyses have been carried out at a single age time point (Lorenzo-Martín et al., 2022), it is still unclear how Vav2 signaling influences the functional state of HFSCs throughout the lifespan of the animals. Here, we have now approached this issue by examining the effect of constitutive Vav2^Onc^ signaling in the transcriptome of HFSCs isolated from six independent age points in mice.

Our data indicate that the constitutive signaling of Vav2^Onc^ has a very profound impact on the overall transcriptome of HFSCs. Thus, we observed much higher numbers of age-regulated genes in *Vav2* ^Onc/Onc^ HFSCs (64.6%) than in WT HFSCs (31.9%) (Figure 1D). Many of these genes represent *de novo* regulatory events that are not found in the age-dependent transcriptome of WT HFSCs (Figure 1E). Perhaps more importantly, we have found a few interesting features of this Vav2^Onc^-regulated transcriptome. On the one hand, and despite the constitutively catalytic activity exhibited by Vav2^Onc^ and the constant expression of its transcript in the time points analyzed in the present study (Supplementary Table S1), we have found that the Vav2^Onc^-regulated transcriptome is still organized in age-dependent waves of gene expression rather than being fixed over time (Figure 1F; Figure 2). On the other hand, we observed that Vav2^onc^ elicits an extensive rewiring of the gene expression waves that are normally observed in HFSCs (Lorenzo-Martín and Bustelo, 2020). This rewiring involves: 1) the age-dependent expression of genes not found in HFSCs (Figure 2; Figure 3); 2) extensive changes in the time-dependent wave position of genes as compared to WT HFSCs (Figure 3); 3) in some cases, changes in the expression pattern of the dynamically regulated genes (e.g., changing from up-to downregulation) (Figure 3); 4) an increased level of co- or anti-regulation of genes belonging to gene signatures associated with hallmark biological processes and HFSC-related states (Figure 4); and 5) a further synchronization in the co- or anti-regulation of independent functional gene signatures in few co-regulated clusters (Figure 5). As a result of this extensive rewiring, very different age-dependent functional states can be recognized in the Vav2^Onc/Onc^ HFSCs as compared to the WT counterparts (Figure 2, see (Lorenzo-Martín and Bustelo, 2020) for WT reference). In general, these states are associated with larger proliferative responses in the very early postnatal phase and more active and enriched signaling/metabolic programs in the subsequent time points analyzed. It is expected therefore that, as is the case of 2.5-month-old mice (Lorenzo-Martín et al., 2022), the HFSCs from older *Vav2* ^Onc/Onc^ mice would have an increased regenerative potential as compared to that of control mice.

It is difficult to decipher the master transcriptional regulators that underlie this extensive transcriptome rewiring using wet-lab approaches. To shed light on this issue, we used *in silico* approaches based on the identification of transcriptional hubs in the Vav2^Onc^-regulated gene expression program and, subsequently, the search for binding sites for those transcriptional hubs in the promoter regions of Vav2^Onc^-regulated genes. Using this approach, we have found that Vav2^Onc^ promotes an extensive rewiring and amplification of the age-dependent transcription factor landscape present in HFSCs (Figure 7). This landscape includes transcription factors of the Cebp, Creb, Ets, Fox, Hox, and Sox families, many of which have been previously linked to the regulation of HFSC identity and homeostasis (Zhan et al., 2009; Lien et al., 2011). Interestingly, these Vav2^Onc^-engaged transcriptional hubs are specifically enriched in quiescent and to a lesser extent active HFSCs, but not in subsequent epidermal cell differentiation stages. In addition to transcriptome hubs whose expression is specifically regulated in *Vav2* ^Onc/Onc^ HFSCs, we have found other hubs that do not undergo statistically significant changes in expression between *Vav2* ^Onc/Onc^ and WT HFSCs (Figure 7D). This suggests that a significant part of the Vav2^Onc^-regulated transcription factor landscape could be regulated by posttranscriptional mechanisms. Future studies using wet-lab approaches will be needed to further validate this potential Vav2^Onc^-regulated transcription factor landscape.

Another important issue that remains unsolved is the most proximal signaling elements that work downstream of Vav2^Onc^ in this process. In terms of the potential GTPase substrates of Vav2^Onc^ involved, we have previously shown that endogenous Vav2^Onc^ triggers the stimulation of Rac1 and, to a lower extent RhoA in primary keratinocytes. By contrast, it cannot stimulate Cdc42 in the same cell system (Lorenzo-Martín et al., 2020a). In agreement with these observations, we could demonstrate using organotypic 3D models that the epithelial hyperplasia induced by Vav2^Onc^-expressing primary keratinocytes is Rac1–Pak1-and RhoA–Rock-dependent (Lorenzo-Martín et al., 2020a). Using an alternative experimental system, we have also demonstrated before that the defective signaling, proliferation, and carcinogen-induced tumorigenesis found in primary *Vav2* ^−/−^;*Vav3* ^−/−^ keratinocytes were connected to reduced activation levels of Rac1 (Menacho-Marquez et al., 2013). All these data suggest that the roles of Vav2 proteins in HFSCs must be Rac1-dependent and, probably, RhoA-dependent as well. As discussed before in the case of Vav2^Onc^, we also consider as highly unlikely that the age-dependent modulation of the Vav2^Onc^-regulated transcriptome would be just the consequence of changes in the expression levels of the transcripts of these two GTPases. Consistent with this idea, we have found that the expression levels of *Rac1* and *Rhoa* mRNAs remain unchanged, with the single exception of wave −12 (in which they are partially downregulated), in most of the time points analyzed in this study (Supplementary Table S1). We cannot rule out at this moment, however, that Vav2^Onc^ and/or the downstream GTPases could undergo cyclic changes at either the translational or posttranslational level that could explain some of the expression waves observed. Likewise, it can be also possible that adaptors functions mediated by the C-terminal Vav2 SH3–SH2–SH3 region (Figure 1A) could contribute in parallel to such functions. Further work will be required to address all these important mechanistic issues.

Data from our previous study (Lorenzo-Martín et al., 2022) and the present work suggest that Vav2^Onc^ will promote increased numbers of HFSCs endowed with more basal regenerative potential for longer periods of time in mice. However, they also give a warning sign, since it is foreseeable that this potentially beneficial feature could represent a problem when oncogenic mutations arise in those cells. In fact, we have previously demonstrated that Vav2^Onc^ promotes the generation of a protumorigenic, hyperplastic niche in the skin that can facilitate tumor formation upon the emergence of genetic lesions in the keratinocyte cell layer of the skin (Lorenzo-Martín et al., 2020a). On the brighter side, it is possible to envision that the Vav2^Onc^-associated transcriptome of HFSCs will have specific Achilles’ heels that can be targeted to hamper the fitness of cancer HFSCs. Such an avenue has been, in fact, demonstrated in the case of more differentiated epithelial components of the skin (Lorenzo-Martín et al., 2020a; Lorenzo-Martín et al., 2020c).

## Data Availability

The datasets presented in this study can be found in online repositories. The names of the repository/repositories and accession number(s) can be found below: NCBI GEO under accessions GSE140152. Publicly available datasets were also analysed and can be found at NCBI GEO under GSE137176.
