# Supplementary figures and images for: The Rho GTPase exchange factor Vav2 promotes extensive age-dependent rewiring of the hair follicle stem cell transcriptome
Source: Front Cell Dev Biol. 2023 Sep 26;11:1252834. doi: 10.3389/fcell.2023.1252834 (PMC10562702; doi:10.3389/fcell.2023.1252834)

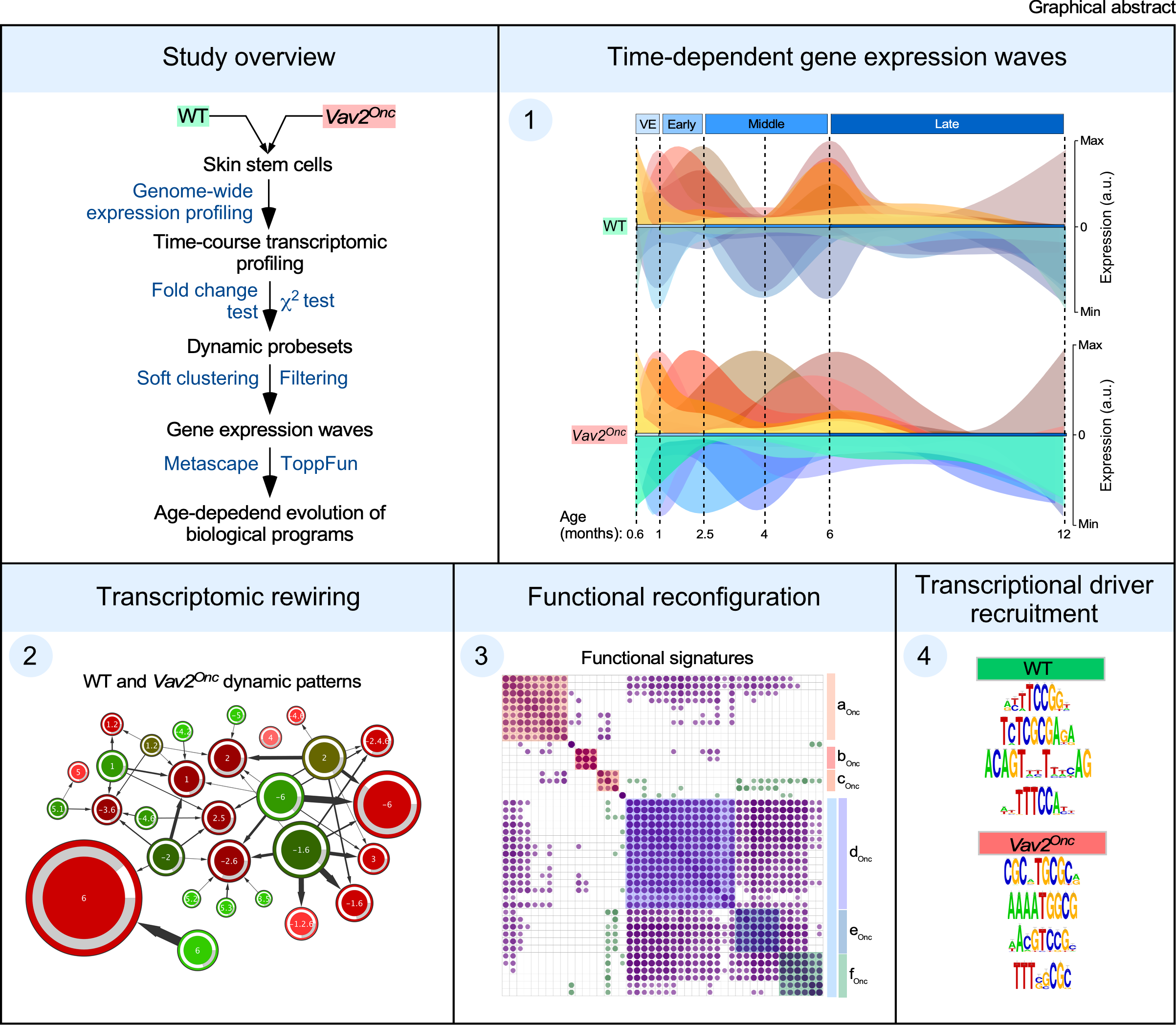

Supplement: Supplementary file 2 [file Image1.TIF]
